# Infants Encode Phonetic Detail during Cross-Situational Word Learning

**DOI:** 10.3389/fpsyg.2016.01419

**Published:** 2016-09-21

**Authors:** Paola Escudero, Karen E. Mulak, Haley A. Vlach

**Affiliations:** ^1^The MARCS Institute for Brain, Behaviour and Development, Western Sydney University, PenrithNSW, Australia; ^2^Centre of Excellence for the Dynamics of Language, Australian Research Council, CanberraACT, Australia; ^3^Department of Educational Psychology, University of Wisconsin, MadisonWI, USA

**Keywords:** lexical development, early word learning, cross-situational learning, statistical learning, minimal pairs, phonetic detail

## Abstract

Infants often hear new words in the context of more than one candidate referent. In cross-situational word learning (XSWL), word-object mappings are determined by tracking co-occurrences of words and candidate referents across multiple learning events. Research demonstrates that infants can learn words in XSWL paradigms, suggesting that it is a viable model of real-world word learning. However, these studies have all presented infants with words that have no or minimal phonological overlap (e.g., BLICKET and GAX). Words often contain some degree of phonological overlap, and it is unknown whether infants can simultaneously encode fine phonological detail while learning words via XSWL. We tested 12-, 15-, 17-, and 20-month-olds’ XSWL of eight words that, when paired, formed non-minimal pairs (MPs; e.g., BON–DEET) or MPs (e.g., BON–TON, DEET–DIT). The results demonstrated that infants are able to learn word-object mappings and encode them with sufficient phonetic detail as to identify words in both non-minimal and MP contexts. Thus, this work suggests that infants are able to simultaneously discriminate phonetic differences between words and map words to referents in an implicit learning paradigm such as XSWL.

## Introduction

Learning new words is a difficult task. In any one moment in time, the world presents learners with a seemingly infinite number of potential referents for just one word (Quine, 1960). However, despite the ambiguity and difficulty of the task, infants are able to acquire new words, with Australian English-learning infants going from saying zero or a few words at 12 months, to over 100 words 8 months later, to over 400 words 8 months after that (Kalashnikova et al., 2016). To understand how infants become such remarkable word learners, much research has examined the cognitive processes that contribute to word mapping.

Word mapping occurs in noisy contexts with many words and potential referents for those words. Research on word mapping has historically focused on how learners resolve ambiguity in word-referent mapping in one moment in time. However, in naturalistic language learning environments, infants must resolve ambiguity across many moments in time. As a result, recent research has shifted toward examining how learners resolve ambiguity across learning events. This work has revealed that learners can track the co-occurrence of words and referents across time and later use this information to infer word-referent mappings. This behavior is commonly termed *cross-situational* or *statistical word learning* (for a review, see Yu and Smith, 2012; also see Smith and Yu, 2008; Frank et al., 2009; Vouloumanos and Werker, 2009; Fazly et al., 2010; Smith et al., 2011; Kachergis et al., 2012; Scott and Fisher, 2012; Suanda and Namy, 2012; Vlach and Johnson, 2013; Yurovsky et al., 2013).

A cross-situational word learning (XSWL) paradigm has been used to model the noisy environments in which infants learn word mappings. In a typical experiment (e.g., Smith and Yu, 2008; Vlach and Johnson, 2013), infants are presented with a series of ambiguous learning trials with two auditory words and two objects. The words and objects are presented in a random order and thus infants are not provided with explicit word-object mappings. After learning, infants are presented with preferential looking test trials in which two objects are presented while one word is presented repeatedly during the trial. If infants look significantly more to the named object relative to the unnamed object, it is assumed that infants have mapped the word to that particular object. The results of these studies have revealed that infants as young as 12 months can infer word-object mappings using XSWL (Smith and Yu, 2008) and that the ability to learn mappings continues to develop across the second year of life (Vouloumanos and Werker, 2009; Vlach and Johnson, 2013).

To date, all XSWL experiments with infants have used novel words that contain little to no phonological overlap, such as the words BLICKET and GAX (e.g., Smith and Yu, 2008; Vlach and Johnson, 2013). Consequently, infants do not need to encode all of the phonetic characteristics of words to distinguish each word from the other words. In some cases (such as with BLICKET and GAX), words can be distinguished by the number of syllables and/or word length alone, requiring minimal to no encoding of phonological detail. However, in real-world situations, infants hear words with varying degrees of phonological overlap, such as words that form minimal word pairs, which are identical except for one consonant, such as BET and PET, or one vowel, such as BET and BIT. Because the extant literature has used words with little to no phonological overlap, we do not know if infants are able to distinguish minimal word pairs during XSWL.

One hypothesis is that infants cannot learn minimal word pairs during XSWL. Adults often experience difficulty learning minimal word pairs in explicit word learning tasks (Papagno and Vallar, 1992; Escudero et al., 2013, 2014b). Moreover, research on infants’ learning of minimal word pairs has suggested that young learners have difficulty encoding phonological detail during word mapping. Although infants are able to discriminate most consonant contrasts from birth, and retain the ability to discriminate the consonant contrasts of their native language at 12 months (but lose the ability to discriminate some non-native contrasts: Werker and Tees, 1983, 1984), this ability does not immediately transfer to explicit word learning. For example, in a Switch task (Stager and Werker, 1997; Werker et al., 1998), 14-month-olds were shown a novel word-object pairing on a screen (e.g., LIF paired with a novel image), until they had become habituated to it, indicated by a criterial reduction in looking time to the pairing. After habituating to the novel word-object pairing, infants were shown the same word-object pairing (Same trial), or the same object paired with a non-minimally different word (NEEM; Switch trial). Infants increased their looking to the Switch trial, indicating that they recognized the difference between LIF and NEEM. However, when presented with the novel minimally different words BIH and DIH, infants did not show an increase in looking to the Switch trial, indicating that they did not notice a difference between the minimal pair (MP). In sum, this work demonstrates that infants struggle to make phonological discriminations while learning word-object mappings, and may be expected to perform poorly at learning minimal word pairs in a XSWL paradigm.

However, other research suggests that infants may be able to make phonological discriminations while successfully learning new words via XSWL. In paradigms where there is more phonetic variability in the learning environment, infants can make fine phonological discriminations (Thiessen, 2007, 2011). Moreover, subsequent research with the Switch task has demonstrated that infants can encode sufficient fine phonological detail to discriminate MPs when provided with additional context. For instance, infants can learn object labels when they are presented in a sentential context or in conjunction with known words (Fennell and Waxman, 2010) or when they are familiarized to the objects prior to test (Fennell, 2012). Fourteen month-old infants demonstrate learning of novel MP words when tested with a preferential looking paradigm of two items (Yoshida et al., 2009), which is the format of the testing phase in the infant XSWL paradigm. Taken together, this work suggests that infants may be able to distinguish minimal word pairs in more naturalistic word learning contexts. XSWL paradigms provide more context and model real-world, noisy environments to a greater degree than previous word mapping tasks (e.g., the Switch task). Indeed, hearing a collection of phonetic structures across learning events may support infants’ ability to simultaneously detect phonetic distinctions and map words to referents.

We tested 12-, 15-, 17-, and 20-month-old infants’ XSWL. The youngest age group (i.e., 12-month-olds) was chosen because this is the youngest age at which infants have been shown to learn cross-situational statistics (Smith and Yu, 2008). Moreover, as outlined above, infants are also able to discriminate native contrasts at 12 months of age (Werker and Tees, 1983, 1984). The remaining age groups were selected to span the typical developmental period examined in research on infants’ MP learning using a looking paradigm (i.e., 14–18 months; e.g., Stager and Werker, 1997; Werker et al., 2002; Thiessen, 2007; Escudero et al., 2014a) and XSWL (i.e., 12–20 months; Smith and Yu, 2008; Vouloumanos and Werker, 2009; Yu and Smith, 2011; Vlach and Johnson, 2013). The goal of sampling these age groups was to test whether infants can simultaneously learn minimal and non-MPs during the period of development that has been of interest to both groups of researchers.

During the training phase of the current experiment, which was a typical XSWL task, infants were exposed to eight novel CVC word-object pairings during a learning phase. In each test trial, infants heard one of the novel words four times and were tested on their ability to look to the object that corresponded to the target word in the context of a distractor object that corresponded to another word from the same set of eight presented during training. Of the eight words, four differed by their initial consonant (BON, DON, PON, TON), and four by their vowel (DEET, DIT, DOOT, DUT). When the two objects presented on the screen referred to two words that differed in only one consonant or one vowel, the trial was a MP (e.g., PON–DON or DEET–DIT), and when the two objects on the screen corresponded to two words that differ in more than one segment, the trial was a non-MP (e.g., BON–DEET). In order to recognize the object that corresponds to the target word in an MP trial, the infant must have encoded fine phonological detail of the words corresponding to both objects to succeed, while less encoding of phonetic detail is required to recognize the corresponding object in a non-MP trial. Having to fully encode phonetic detail for words that form MPs makes our task substantially more difficult than the task posed in previous XSWL studies, and disambiguation of even our non-MPs is expected to be more difficult because all words were in the same syllabic context, namely monosyllables in the consonant-vowel-consonant (CVC) context. The use of a CVC context restricted the differences between words to a relatively small set of segmental distinctions, whereas the words included in previous studies differed in both segments and number of syllables (e.g., DAX-BLICKET; Vlach and Johnson, 2013) or included two-syllable words with more segmental differences (e.g., MANU-COLLAT; Yu and Smith, 2011).

We predicted that despite the increased difficulty posed by our chosen non-MP words, infants would be able to identify words in a non-MP context (e.g., identify BON when the distractor image is DEET), based on previous research demonstrating that 12- to 20-month-old infants can learn non-MP words during XSWL (Smith and Yu, 2008; Vlach and Johnson, 2013). We hypothesized that infants would experience more difficulty identifying target words in an MP context which would require encoding words with fine phonetic detail, with performance in an MP context perhaps not exceeding chance. This is because infants often fail to encode phonetic detail in other word learning tasks, such as the Switch task (Stager and Werker, 1997; Werker et al., 1998; Escudero et al., 2014a). Moreover, even when older children (5- to 7-year-olds) demonstrate rapid learning of words that form MPs, they have lower accuracy and slower reaction times for them when compared to non-MPs (Giezen et al., 2016).

## Materials and Methods

### Participants

Participants were twenty-two 12-month-olds (*M* = 12.3 mos, range = 11.5–13.0 mos, nine females), twenty-four 15-month-olds (*M* = 14.8 mos, range = 13.8–15.6 mos, 13 females), twenty-six 17-month-olds (*M* = 17.3 mos, range = 15.9–18.2 mos, 10 females), and sixteen 20-month-olds (*M* = 19.7 mos, range = 18.6–20.6 mos, eight females), resulting in 88 participants in total. Enrollment in the study was open until all age groups consisted of at least 16 participants or reached 30 participants, whichever occurred first. These numbers were chosen based on previous research demonstrating that a sample size of 16 infants is of sufficient power for detecting learning (e.g., Vlach and Johnson, 2013) and to reflect the sample sizes typically used in infant XSWL studies (i.e., 16–30 participants in each age group; Smith and Yu, 2008, 2013; Vouloumanos and Werker, 2009; Yu and Smith, 2011; Vlach and Johnson, 2013).

Infants’ caregivers provided written, informed consent in accordance with the Western Sydney University Human Research Ethics Committee. The infants were primarily Caucasian from middle- to upper-middle-class households in Sydney, Australia. Another 32 infants were excluded from the final sample due to fussiness (*N*_12mos_ = 7; *N*_15mos_ = 2; *N*_17mos_ = 2, *N*_20 mos_ = 4), failure to look at either image for a minimum of 200 ms during the 1500 ms analysis window for at least one non-MP trial and one MP trial (*N*_12mos_ = 4; *N*_15mos_ = 4, *N*_17mos_ = 3, *N*_20mos_ = 3), or experimenter error (*N* = 3).

### Stimuli

#### Novel Words

**FIGURE 1:**
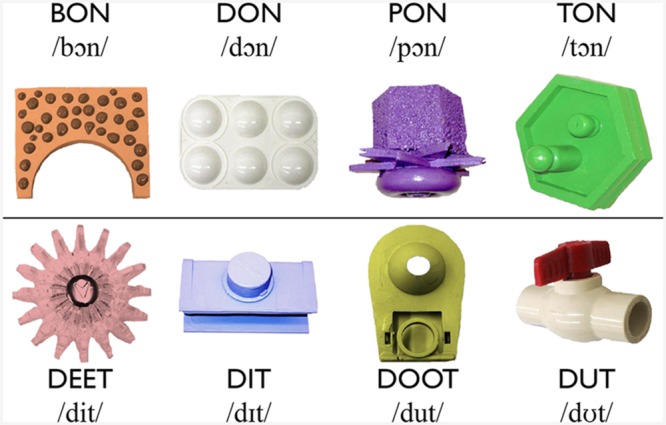
**The eight novel words and their visual referents.** The vowel used for the consonant minimal pairs (MPs) in the top row is /ᴐ/ as in POT. The vowels used in the vowel MPs are /i/ as in BEAT, /ɪ/ as in BIT, /u/ as in BOOT, and /Ʊ/ as in PUT.

Eight monosyllabic nonsense words were recorded by a female native speaker of Australian English. As shown in **Figure 1**, the words followed a CVC structure, adhered to English phonotactics, and have been used in previous research on the acquisition of MPs (Curtin et al., 2009; Fikkert, 2010). Four of the words differed minimally in their first consonant, whereas the other four differed in their vowel. Two tokens of each of the eight spoken words were selected for use in the experiment so that intonation contours were comparable across words.

#### Novel Visual Referents

Each nonsense word was randomly paired with a visual referent (see **Figure 1**). The same word-referent pairings were presented to all participants. The visual referents for the words were pictures of novel items used in previous studies on XSWL (Vlach and Sandhofer, 2014). Each image measured 280 × 274 pixels. Slides were created in which two of the eight visual referents were placed on an 800 × 600-pixel white background with the top-left corner of the left image positioned at 20 × 163 pixels, and the top-left corner of the right image positioned at 500 × 163 pixels. Two areas of interest (AOIs) were defined around the left- and right-positioned novel visual referents. Each AOI measured 360 × 320 pixels, with the top left corner of the left AOI positioned at 0 × 120 pixels, and the top left corner of the right AOI positioned at 480 × 120. The same AOIs were used for all trials.

#### Attention Videos

Each attention video consisted of a looped cartoon animation measuring 170 × 170 pixels, which was centered on the monitor between every third trial in the learning phase and between each trial in the testing phase. Each animation was paired with a non-linguistic sound.

### Procedure

Participants’ gaze was measured for the duration of the experiment using a Tobii X120 eye tracker (Tobii Technology, Danderyd, Sweden) sampling at 60 Hz.

**FIGURE 2:**
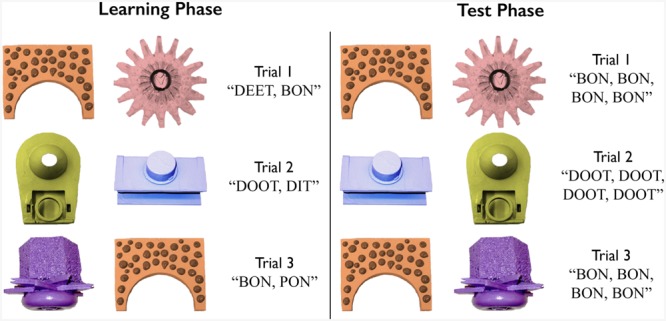
**Examples of learning and test trials**.

The experiment consisted of a learning phase and testing phase. Examples of learning and testing phase trials can be seen in **Figure 2.** At the beginning of the experiment, participants were seated on their caregiver’s lap in front of a 17-in. monitor so that their eyes were 70 cm from the front of the eye tracker. For the duration of the study, caregivers wore MTUNE headphones (Macally, Ontario, CA, USA) that played a mixture of music and speech, and were instructed to look down or to the side during the experiment. This kept caregivers blind to the experiment, and also served to assure that it was the children’s and not the caregivers’ gaze that was tracked. Before testing began, participants’ gaze was calibrated to a nine-point grid on the screen. The calibration, learning and testing phases were presented using Tobii Studio version 2.0.8 (Tobii Technology, Danderyd, Sweden).

#### Learning Phase

The learning phase consisted of 36 trials. Across the 36 trials, participants were presented with each word-referent pair nine times. In each learning trial, two of the eight visual referents displayed on the screen. After 500 ms, the word corresponding to each item was spoken so that each of the two images was named once, either left to right, or right to left, with 500 ms of silence between spoken words. There was no indication of the order in which the visual referents were named.

The presentation order of the paired trials was randomized for each participant and the pairings were controlled such that each visual referent occurred with every other visual referent at least once, and no more than twice. If the same pairing occurred more than once, the designations of the left and right image were swapped so that participants never saw the exact same visual pairing more than once. As each word appeared nine times, the occurrence of an image in the left or right position was balanced such that half of the images appeared five times on the left and four times on the right, while the other half appeared in the opposite pattern. Whether a visual referent was named first or second, and the number of times each of the two tokens of each nonsense word were heard, were balanced similarly.

The pair in each learning trial formed either a non-MP, in which two or three segments differed between words (e.g., BON–DEET or DON–DEET), or a MP in which only one segment differed between words. The MPs were either consonant MPs (e.g., BON–TON) or vowel MPs (e.g., DEET–DIT). In total, the learning set consisted of 24 non-MPs and 12 MPs, split evenly between consonant and vowel MPs. Each learning trial lasted 3.5 s and the attention getters, presented between every three learning trials, played until participants’ gaze was centrally fixed. The total duration of the learning phase was approximately 3 min.

#### Test Phase

**FIGURE 3:**
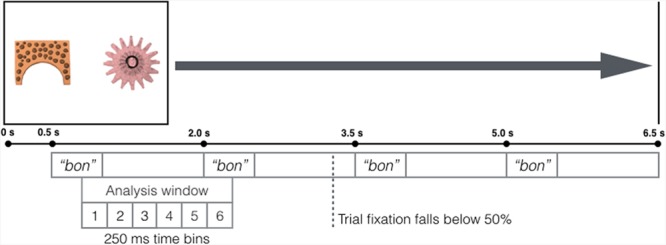
**Layout of test trials**. After two images were displayed for 500 ms, participants heard four repetitions of the auditory label corresponding to one of the images. Our analysis window comprised six 250 ms time bins which began 301 ms after onset of the first word token, and corresponded to the first word token and its interstimulus interval.

A schematic representation of a test trial is given in **Figure 3.** The test phase immediately followed the learning phase. There were three between-subjects test conditions. Each condition comprised 12 of the 36 image pairings from the learning phase, such that during test, infants saw 12 of the 36 visual referent pairings they had seen during training, though the left-right designation of the images in each pair was randomized once. As in the learning phase, participants viewed trials in which two images were presented side-by-side. Unlike in the training phase, where participants heard the auditory label referring to each image, for each test trial, once the two images had been on the screen for 500 ms, the spoken word corresponding to just one of the images (the target object) played four times. This word was presented in two alternating repetitions of the two tokens, with at least 500 ms between each repetition such that the onset of each repetition began exactly 1.5 s after the onset of the previous repetition. Each word used in the experiment served as the target in the test phase at least once, and no more than twice. During the trial, participants’ gaze to the target and distractor images was recorded. The testing phase consisted of eight non-MP trials and four MP trials (two consonant and two vowel MP trials). Each trial lasted 6.5 s, and attention getters played at the beginning of each trial until participants’ gaze was oriented to the center of the screen. The total duration of the testing phase was approximately 2 min.

## Results

Raw gaze points were converted to fixations by applying the Tobii Fixation Filter (an implementation of Olsson, 2007, pp. 37–40) with a fixation radius of 35 pixels. Analysis of learning phase trials began 301 ms after onset of the initial word token to account for the conservative estimate of the minimal time to process an auditory stimulus (100 ms) and carry out a fixation (200 ms) in reaction to the presentation of the first word token (Salthouse and Ellis, 1980). Similarly, learning trials that did not have a minimum 200 ms fixation time to either the target or distractor image from 301 ms after initial word onset until the end of the trial were removed from analysis. On average, participants fixated to 32.4 of 36 training trials (*SD* = 3.9), and this did not differ across age groups (*F*[3, 87] = 1.25, *p* = 0.296).

**FIGURE 4:**
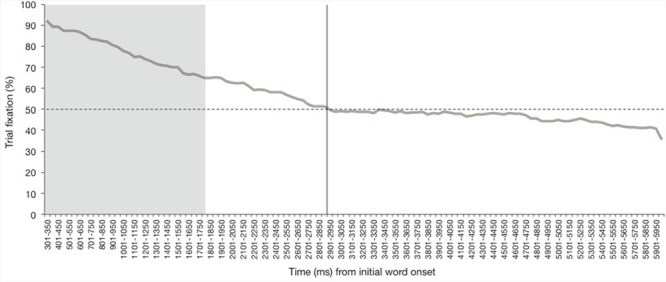
**Percent fixation to the test trials (i.e., fixation to either the target *or* distractor image).** The vertical line indicates the point at which trial fixation dropped below 50%. The shaded area represents the chosen window of analysis.

We next plotted infants’ overall fixation time during test trials in order to assess attention during the test trial and select our window of analysis (**Figure 4**). Fixation data for each participant was divided into 50 ms bins, beginning 301 ms after onset of the first word token. Average trial fixation (i.e., the percentage of time spent fixated to *either* the target or distractor image) was calculated for each bin. Participants’ fixation to individual test trials decreased as the trial went on. At the time bin corresponding to 2851–2900 ms after initial word onset, fixation to either image in the test trial dropped below 50%, and did not recover to above 50% again (as indicated by the vertical line in **Figure 4**). This means that on average, over half of participants did not fixate to the trial past this point in time. This drop-off point occurs after the onset of the second word token, but before the onset of the third word token. Because looking remained above 50% only for the first word and interstimulus interval segment, we focused our analysis window there. This resulted in a 1.5 s analysis window, which encompasses the shaded region on **Figure 4.** As explained above, and as can be seen in **Figure 3**, the analysis window was shifted by 300 ms so that it began 301 ms after onset of the first word, and ended 1800 ms after onset of the first word, or 300 ms after the onset of the second token. Due to the conservative estimate of the physiological minimum time to process auditory stimulus (100 ms) and carry out a responding fixation (200 ms; Salthouse and Ellis, 1980), fixation during the first 300 ms post-word onset cannot be attributed to that word, and therefore fixation from 1 to 300 ms after onset of the second word token is attributed to the first word token. Notably, the window of analysis used here corresponds almost precisely to that used by Fernald et al. (2001) to investigate infant word recognition, and corresponds generally to other timecourse analyses of infant word recognition that have used windows of analysis that are no more than 2 s post initial word onset in length (Swingley et al., 1999; Zangl et al., 2005), as looking behavior by infants beyond that point is thought to no longer be related to the auditory stimulus (Swingley et al., 1998).

Test trials that did not have a minimum 200 ms fixation time to either the target or distractor during the window of analysis were removed. Participants fixated on 6.5 (*SD* = 1.7) of eight non-MP trials and 3.3 of 4 MP trials (*SD* = 0.8), and this did not differ across age groups (non-MP: *F*[3, 87] = 0.67, *p* = 0.573; MP: *F*[3, 87] = 0.39, *p* = 0.763).

**FIGURE 5:**
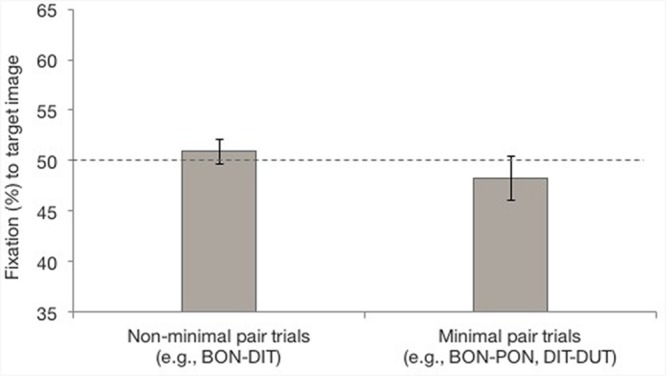
**Percent fixation to the target image for non-MP and MP test trials, starting from 301 ms to 1800 ms after onset of the first word.** Error bars represent one standard error.

To determine whether fixation to targets differed for non-MP versus MP trials and whether performance differed across age groups, participants’ percent looking to the target image was analyzed in a linear mixed-effects model with pair type (non-MP, MP) and age group (12, 15, 17, and 20 months) entered as fixed factors, and subject, target, distractor image, and order entered as random effects. Notably, with this type of analysis, non-systematic differences in sample sizes across groups do not influence the model’s outcome (Pinheiro and Bates, 2000). The model revealed no effect of pair type [*χ*^2^(1, *n* = 857) = 0.05, *p* = 0.818] or age group [*χ*^2^(3, *n* = 857) = 1.68, *p* = 0.641], and no interaction between the two [*χ*^2^(3, *n* = 857) = 3.06, *p* = 0.382]. Further, percent fixation to the target image did not differ from chance for either pair type (non-MP: *t*(87) = 0.72, *p* = 0.474, 95% CI [-1.57%, 3.35%]; MP: *t*(87) = 0.58, *p* = 0.564, [-3.00%, 5.46%]). **Figure 5** shows infants’ overall percent fixation to the target image for the duration of the window of analysis.

**FIGURE 6:**
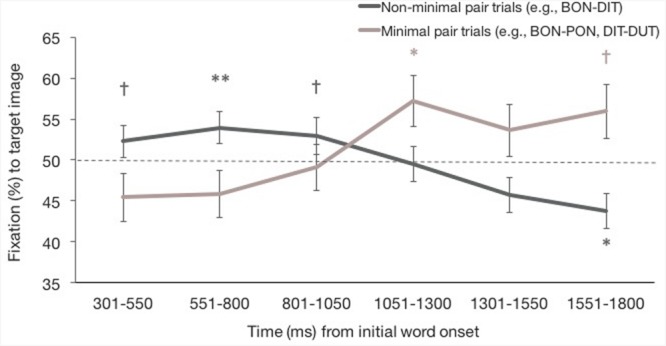
**Percent fixation to the target image for non-MP and MP test trials for the six 250 ms time bins beginning from 301 ms after onset of the first word token.** Error bars represent one standard error. ^∗∗^*p* < 0.01, ^∗^*p* < 0.05, ^†^*p* < 0.01.

While this initial analysis suggests that infants did not learn words via XSWL, previous studies of XSWL have found that performance varies across time (e.g., Vouloumanos and Werker, 2009; Yu and Smith, 2011). That is, gaze behaviors during eye-tracking experiments should be thought of as dynamic, rather than static, and as a result, there are often windows of time in which infants demonstrate learning or recognition, rather than across the entire trial duration. This has been demonstrated by Richmond and Nelson (2009) and Richmond and Power (2014), who have shown that analyzing infants’ looking patterns in second-long segments obscures looking behavior that emerges when analyzing looking behavior across 250 ms segments. Thus, we conducted a time course analysis in line with this approach. Our total window of analysis was divided into six 250 ms time bins. The percentage of fixation time to the target image during test trials across these 250 time segments was calculated by summing the fixation time that fell within the target image during each time bin, and dividing this by the total fixation time falling within the target and distractor images during the 250 ms. The average target fixation for each time segment for both pair types can be seen in **Figure 6.**

Results from the model show that there was no main effect of age group [*χ*^2^(3, *n* = 4515) = 0.46, *p* = 0.929], and no interactions involving age group [age group x pair type: *χ*^2^(3, *n* = 4515) = 1.69, *p* = 0.640; age group x time bin: *χ*^2^(15, *n* = 4515) = 9.87, *p* = 0.828; age group x pair type x time bin: *χ*^2^(15, *n* = 4515) = 18.22, *p* = 0.251], though **Table 1** shows infants’ percent target fixation for each time bin, for each age group and for non-MP and MP trials. There was also no main effect of pair type [*χ*^2^(1, *n* = 4515) = 0.01, *p* = 0.910] or time bin [*χ*^2^(5, *n* = 4515) = 5.02, *p* = 0.413]. However, the interaction of pair type and time bin was significant [*χ*^2^(5, *n* = 4515) = 13.90, *p* = 0.016]. For non-MPs, fixation to the target image decreased between the third and fourth time bin (*p* = 0.038, 95% CI [-6.98%, -0.20%]). For MPs, target fixation increased between the third and fourth time bin (*p* = 0.040, [0.26%, 11.07%]). Further, percent target fixation was greater for non-MPs than MPs during the first (*p* = 0.024, [1.02%, 14.28%]) and second (*p* = 0.026, [0.90%, 14.17%]) time bin, but this pattern reversed for the sixth time bin (*p* = 0.030, [-15.12%, -0.79%]), such that percent target fixation was greater for MPs.

**Table 1:** Mean percent fixation to the target image for non-minimal pair (non-MP; e.g., BON–DIT) and minimal pair (MP; e.g., BON–TON, DIT–DUT) test trials by age group. Data are divided into six 250 ms time bins beginning 301 ms after onset of the first word token, and ending 1800 ms after onset of the first token.

	301–550 ms		551–800 ms		801–1050 ms		1051–1300 ms		1301–1550 ms		1551–1800 ms	
	Non-MP	MP	Non-MP	MP	Non-MP	MP	Non-MP	MP	Non-MP	MP	Non-MP	MP
12 months	50.61 *(19.98)*	48.59 *(30.85)*	55.95 *(17.78)*	50.39 *(35.58)*	57.58 *(22.51)*	47.11 *(34.08)*	50.42 *(22.70)*	51.78 *(31.03)*	37.80 *(22.79)*	48.54 *(32.46)*	37.27 *(18.53)*	55.38 *(28.95)*
15 months	51.53 *(18.11)*	55.19 *(30.10)*	56.64 *(20.99)*	51.01 *(23.78)*	53.84 *(26.49)*	52.03 *(29.93)*	51.78 *(21.48)*	60.72 *(31.66)*	45.91 *(25.51)*	60.82 *(33.82)*	43.67 *(23.86)*	61.88 *(35.82)*
17 months	59.33 *(15.99)*	41.04 *(28.85)*	54.05 *(15.22)*	45.31 *(27.39)*	48.04 *(16.54)*	51.85 *(24.58)*	48.14 *(17.94)*	61.07 *(30.21)*	52.37 *(15.27)*	50.50 *(30.72)*	49.47 *(19.97)*	49.98 *(35.62)*
20 months	52.39 *(19.99)*	36.08 *(28.99)*	55.75 *(19.00)*	36.83 *(29.31)*	60.38 *(21.83)*	48.17 *(25.20)*	53.83 *(23.87)*	59.54 *(34.09)*	51.65 *(18.84)*	60.47 *(31.55)*	48.97 *(23.40)*	63.88 *(26.33)*

*Values in italics represent one standard deviation from the mean.*

As can be seen in **Figure 6**, one-sample *t*-tests against chance performance (50%) showed that for non-MP trials, fixation to the target image was marginally above chance during the first time bin (*t*[87] = 1.92, *p* = 0.059, [-0.14%, 7.66%]), was significantly above chance during the second time bin (*t*[86] = 2.88, *p* = 0.005, [1.71%, 9.35%]), and was again marginally above chance in the third time bin (*t*[87] = 1.80, *p* = 0.075, [-0.44%, 8.34%]). Target fixation did not differ from chance during the fourth (*t*[83] = 0.33, *p* = 0.742, [-3.71%, 5.19%]), and fifth (*t*[87] = -1.39, *p* = 0.169, [-7.71%, 1.38%]), time bins, and was below chance during the sixth time bin (*t*[86] = -2.26, *p* = 0.026, [-9.83%, -0.64%]), indicating fixation to the distractor image. For MP trials, fixation to the target did not differ from chance during the first three time bins (first: *t*[87] = -1.21, *p* = 0.231, [-10.24%, 2.51%]; second: *t*[87] = -1.10, *p* = 0.275, [-9.57%, 2.75%]; third: *t*[87] = 0.15, *p* = 0.988, [-5.97%, 6.06%]), but was above chance during the fourth time bin (*t*[86] = 2.50, *p* = 0.015, [1.70%, 15.00%]). Target fixation did not differ from chance during the fifth time bin (*t*[84] = 1.31, *p* = 0.192, [-2.34%, 11.48%]), but was marginally above chance during the sixth time bin (*t*[82] = 1.98, *p* = 0.051, [-0.02%, 14.10%]).

## Discussion

We examined whether infants can simultaneously discriminate phonological differences among words and learn word mappings during XSWL. The results indicate that infants learned word mappings with sufficient phonological detail as to identify words in non-minimally different word pairs (non-MPs). During non-MP test trials, infants showed a looking preference to the target image during the second time bin and marginal looking preference to the target image during the first and third time bins. Though distinguishing words in a non-MP context does not require much fine phonetic encoding, this experiment carried increased demand relative to other studies of XSWL by infants at this age (Smith and Yu, 2008; Yu and Smith, 2011; Vlach and Johnson, 2013), as all words in the present study had a CVC structure and were monosyllabic. Thus, even for the non-MP test trials, infants had fewer cues than in previous studies by which to differentiate words, but were still able to learn word-object pairings in a non-MP context. The results also demonstrate that infants can learn minimally different word pairs (MPs) via XSWL: Infants showed a looking preference to the target image during the fourth time bin for MPs, and marginal looking preference to the target image during the sixth time bin. This finding contrasts with the complete failure for learning MPs reported in most previous early word learning studies that use explicit word learning paradigms and looking times for the whole trial, such as the Switch task.

Overall, the results of the present study demonstrate that infants can learn words during XSWL, even when phonetic discrimination and encoding of words is more challenging. Therefore, it contributes to the growing body of research demonstrating that XSWL is a viable means by which infants resolve referential ambiguity and acquire word-referent mappings (Smith and Yu, 2008; Vouloumanos and Werker, 2009; Vlach and Johnson, 2013).

Although this research provides evidence that infants can learn non-MPs and MPs during XSWL, there are limitations in interpreting the results. Thus, this work should be viewed as a first step in understanding infants’ phonological processing during XSWL. For instance, it is unclear why infants fixated on the target at the beginning of the test trial for non-MPs, whereas there was a delay to target fixation at test for MPs. The slight delay in target fixation for MPs relative to non-MPs may be due to the fact that vowel MPs (i.e., half of MP trials) were differentiated on their second segment (e.g., DIT–DUT), whereas most non-MPs were differentiated in their initial segment. We chose to use stimuli that had been successfully used in previous studies of infant MP learning, and thus our experiment was not designed to test this possibility. Future research could determine whether this apparent delay is due to appearance of the differentiating segment, or perhaps due to differences in processing of consonant MPs versus vowel MPs. It is also unclear why for non-MP trials, infants transition from an initial looking preference for the target item to a preference for the distractor item in the sixth time bin. Future research could test whether this looking behavior relates to processing differences between non-MP and MP trials. Here, we take infants’ target fixation for both non-MP and MP trials as evidence of successful learning of both pair types.

Research in our laboratory has previously demonstrated that adults trained in the same XSWL task (Escudero et al., 2016a) with the same stimuli (Escudero et al., 2016b) learn non-MPs and MPs, with performance being much stronger than the infants observed in the current experiment. Taken together, this work suggests that there are likely to be improvements between infancy and adulthood in the ability to simultaneously encode phonological detail and map words to objects in XSWL. The central goal of the current study was not to outline developmental differences in performance. However, future research should examine developmental improvements in this task. It may be that with much larger samples of infants at each age group, small differences in performance may emerge. Alternatively, it may be that infants begin to demonstrate improvements in encoding phonological detail and word mapping during XSWL after 20 months of age.

Our findings have implications for the role of phonological overlap in word learning. Children’s lexicons contain fewer phonological neighbors (i.e., words that are differentiated by a single phoneme) compared to adults (Charles-Luce and Luce, 1990). Given that there is no straightforward relationship between lexicon size and phonological neighborhood density (Charles-Luce and Luce, 1990, 1995), children’s word representations may be optimized to include only as much phonological detail as is necessary to distinguish phonological neighbors (see also Brown and Matthews, 1997; Metsala and Walley, 1998). While research on this topic has typically looked at the lexicons of pre-school- to elementary-school-aged children, our findings suggest that a lexicon containing sparsely populated phonological neighborhoods is not due to difficulty with encoding fine phonological detail, as here infants encoded newly learned words with fine phonological detail in an implicit learning paradigm, a finding that has been previously reported in explicit word learning tasks (e.g., Werker et al., 2002; Curtin et al., 2009; Escudero et al., 2014a). More recent examination of children’s lexicons has shown that their lexicons contain larger phonological neighborhood densities than previously thought, and that phonological overlap may in fact facilitate children’s word learning (Storkel and Rogers, 2000; Storkel, 2001).

Future work should continue to examine infants’ ability to learn MPs during XSWL. In particular, researchers should examine the mechanisms that lead to constraints on infants’ XSWL learning of non-MP and MP words. For instance, it may be that simultaneously discriminating phonological differences among words while determining word-referent mappings imposes a large cognitive demand on infants. If cognitive demands are minimized, infants may demonstrate less difficulty in learning words. Research using the Switch task supports this proposal: Presenting words in a neutral carrier sentence (e.g., “Look at the BON!”) or providing pre-test trials with familiar items prior to test to reinforce the task objective (see Fennell and Waxman, 2010) can help infants learn MP words. This latter case would make results even more comparable to Escudero et al. (2016b), as adults were tested in a forced-choice task and by necessity were provided with task instructions (though they were not given instruction in the learning phase).

It is possible that differences in performance exist across MPs depending upon the type of phonological detail that must have been encoded in order to identify the target word. However, as our goal was to test children’s overall ability to encode phonological detail in XSWL, we tested infants on a variety of consonant and vowels MPs. Thus, the small sample of trials completed by each participant (inherent to infant behavioral research) was insufficient for direct statistical comparisons among these phonetic features to be made. Future research should directly test how the type of phonetic detail influences early word learning in an XSWL paradigm. For instance, in an experiment designed to test consonant and vowel MPs separately, infants may more readily learn vowel MPs than consonant MPs, as previous experiments using the Switch task have found more failure for consonant MPs (Stager and Werker, 1997; Werker et al., 2002; Pater et al., 2004) than for vowel MPs (Curtin et al., 2009; Escudero et al., 2014a). Similarly, infants may perform differently depending on the specific features that need to be encoded. For instance, Curtin et al. (2009) found that infants could learn a vowel MP differentiated by vowel height (DEET–DIT), but not one that was differentiated by backness (DEET–DOOT), or height and backness (DIT–DOOT).

Another important modification to the current task might be to present more tokens of words that introduce more non-linguistic variation, by presenting tokens spoken by multiple speakers (Rost and McMurray, 2009, 2010) or perhaps by the same speaker in different affects (Singh, 2008). Adding this type of variation has been shown to improve 14-month-olds’ ability to discriminate MPs in the Switch task (Singh, 2008; Rost and McMurray, 2009, 2010). Introduction of more variation, such as variation not critical for discriminating the contrasts, may enable infants to focus on the relatively invariant segmental variation which differentiates the contrast (Rost and McMurray, 2009, 2010). Additionally, to more closely approximate natural word learning (in which MPs are less frequent than non-MPs), the present study presented infants with half as many MP learning trials as non-MP learning trials. Future research should investigate whether increased exposure to MP word learning trials in XSWL results in improved phonological encoding of words.

In sum, this study demonstrates that infants are able to accomplish the difficult task of word mapping in an implicit word learning paradigm. Indeed, infants in the current experiment demonstrated some ability to encode phonological detail during XSWL, allowing them to discriminate and map words to referents in a MP context. Future research should continue to consider the role of infants’ phonological encoding of words in the processes underlying their ability to resolve ambiguity in the world.

## Author Contributions

PE conceived the research project. PE, KM, and HV designed the experiment, KM set up the experiment. KM and HV analyzed the data. All authors wrote the paper.

## Conflict of Interest Statement

The authors declare that the research was conducted in the absence of any commercial or financial relationships that could be construed as a potential conflict of interest.
